# Factors influencing clinical outcomes in patients with diabetic macular edema treated with intravitreal ranibizumab: comparison between responder and non-responder cases

**DOI:** 10.1038/s41598-019-47241-1

**Published:** 2019-07-29

**Authors:** Yen-Po Chen, Ai-Ling Wu, Chih-Chun Chuang, San-Ni Chen

**Affiliations:** 1 Department of Ophthalmology, Changhua Christian Hospital, Changhua, Taiwan; 2 Department of Ophthalmology, Chang Gung Memorial Hospital, Taoyuan, Taiwan; 3 College of Medicine, Chang Gung University, Taoyuan, Taiwan; 4 College of Medicine, Chung-Shan Medical University, Taichung, Taiwan; 5 College of Medicine, Kaohsiung Medical University, Kaohsiung, Taiwan; 6 Department of Optometry, College of Nursing and Health Sciences, Da-Yeh University, Changhua, Taiwan

**Keywords:** Outcomes research, Retinal diseases, Retinal diseases, Outcomes research

## Abstract

Diabetic macular edema (DME) is the leading cause of visual impairment in patients with diabetes mellitus. A retrospective study was conducted to investigate the factors influencing the clinical outcomes in 73 patients (94 eyes) with DME treated with intravitreal ranibizumab therapy. Baseline demographic, systemic, and ocular data were assessed for the association with visual and anatomic outcomes after treatment. The mean best corrected visual acuity (BCVA) improved from 0.92 ± 0.45 to 0.61 ± 0.43 logarithm of the minimum angle of resolution (LogMAR) (p < 0.001) after treatment. The mean central subfield macular thickness (CST) decreased from 425.2 ± 127.4 to 328.6 ± 99.4 μm (p < 0.001). The treatment response was significantly influenced by Age (p = 0.003) and baseline BCVA (p = 0.001). In addition, glycosylated hemoglobin (HbA1c) (p = 0.013) and proliferative diabetic retinopathy (PDR) (p = 0.019) were the prognostic factors for the visual outcome in the responders and non-responders, respectively. Moreover, baseline CST was the strongest predictor of anatomic outcome in all subjects (p < 0.001). Intravitreal ranibizumab for DME resulted in significant improvement in clinical outcomes. Younger age and better baseline BCVA were associated with better visual outcome after the treatment. In addition, glycemic control in the treatment of patients with DME is crucial to achieve better visual outcomes, especially in the responders to ranibizumab treatment.

## Introduction

Diabetic retinopathy (DR) is one of the major causes of legal blindness in adults of working age worldwide^1–3^. Diabetic macular edema (DME) is a leading cause of central vision impairment among patients with DR^4^. The risk for developing DME is associated with longer duration of diabetes and elevated levels of glycosylated hemoglobin (HbA1c)^5^. The global prevalence of DME is estimated to be 7.5%, affecting approximately 21 million individuals^2^. As the prevalence of diabetes is steadily increasing and expected to rise by more than 50% globally from 2000 to 2030, with the number of diabetes cases estimated to reach 366 million worldwide by 2030^6^, DME will therefore causes a tremendous medical burden globally.

Although the pathogenesis of DME has not yet been completely clarified, elevated vitreous levels of vascular endothelial growth factor (VEGF) with increasing vascular permeability is known to play a role in the development of DME^4,7^. In addition, intravitreal anti-VEGF therapy has shown the promising results for treating DME in several large randomized clinical trials recently^8–12^. Intravitreal injection of anti-VEGF agents has become the first-line treatment for patients with DME. However, some studies have shown that there are still some patients who respond poorly to anti-VEGF therapies even after 3 or more injections of anti-VEGF agents^13,14^. A few studies have revealed some factors influencing the clinical outcomes of DME treating by anti-VEGF agents^15–19^, whereas the results are inconsistent among these studies and might not be reliably used in clinical practice.

Thus, the purpose of this study is to identify the factors which influence the visual and anatomic outcomes after intravitreal ranibizumab treatment in patients with DME. Furthermore, the analyses were also applied to investigate the differences in clinical characteristics and prognostic factors between the responder and non-responder cases.

## Results

A total of 94 eyes of 73 patients were included in this study. Thirty-eight (52.1%) were male and 35 (47.9%) were female. The mean age of the patients was 61.2 years (range from 32 to 78). All patients were diagnosed with type 2 DM with mean HbA1c of 7.78 ± 1.32%. Pan-retinal photocoagulation had been performed in 32 eyes with proliferative diabetic retinopathy (PDR) (34.0%). Four eyes with DME (4.3%) had been treated with micropulse laser during the follow-up period. After intravitreal ranibizumab for DME, mean best-corrected visual acuity (BCVA) improved significantly from 0.92 ± 0.45 LogMAR at baseline to 0.61 ± 0.43 LogMAR at final visit (p < 0.001). In addition, mean central subfield macular thickness (CST) decreased significantly from 425.2 ± 127.4 μm to 328 ± 99.4 μm (p < 0.001). The mean number of intravitreal anti-VEGF injection was 9.2 ± 3.6 within the mean follow up time of 22.5 ± 6.5 months.

### Comparison of clinical characteristics between responders and non-responders to intravitreal ranibizumab

Among the 94 eyes, 55 eyes had an improvement of one or more lines in final BCVA following intravitreal ranibizumab therapy and were placed in the responder group. The other 39 eyes were classified into the non-responder group. The baseline characteristics and clinical outcomes according to the response to intravitreal ranibizumab therapy are summarized in Table 1. Patients in the responder group were significantly younger (mean age, 58.3 ± 10.3 years) than those in the non-responder group (mean age, 65.1 ± 8.8 years; P = 0.004). Although the eyes in the responder group had significantly worse BCVA and thicker CST than that of the patients in the non-responder group at baseline (mean 1.08 ± 0.39 vs. 0.68 ± 0.43 LogMAR; p < 0.001 and 425.2 ± 127.4 vs. 394.1 ± 116.0 μm; P = 0.032 for the BCVA and CST, respectively), the eyes in the responder group had significantly better BCVA and thinner CST at final visit (mean 0.44 ± 0.25 vs. 0.86 ± 0.50 LogMAR; p < 0.001 and 310.4 ± 86.8 vs. 354.2 ± 110.9 μm; P = 0.033 for the BCVA and CST, respectively). However, there was no significant difference in HbA1c (7.66 ± 1.40 vs. 7.94 ± 1.21%; p = 0.0.359) and the number of intravitreal injections (9.51 ± 3.68 vs. 8.87 ± 3.37; p = 0.421) between the two groups (Table 1).

**Table 1:** Baseline characteristics and treatment outcomes according to the responder and non-responder groups.

	Total subjects (N = 94)	Responder group (N = 55)	Non-responder group (N = 39)	P value
Age (years)	61.2 (±10.2)	58.3 (±10.3)	65.1 (±8.8)	0.004*
Gender (Male: Female)	49:45	31:24	18:21	0.379
HbA1C (%)	7.78 (±1.32)	7.66 (±1.40)	7.94 (±1.21)	0.359
PDR	32 (34%)	22 (40%)	10 (25.6%)	0.130
Baseline BCVA (LogMAR)	0.92 (±0.45)	1.08 (±0.39)	0.68 (±0.43)	<0.001*
Final BCVA (LogMAR)	0.62 (±0.43)	0.44 (±0.25)	0.86 (±0.50)	<0.001*
Changes in BCVA (LogMAR)	0.30 (±0.53)	0.64 (±0.39)	−0.18 (±0.22)	<0.001*
Baseline CST (μm)	425.2 (±127.4)	447.2 (±131.5)	394.1 (±116.0)	0.032*
Final CST (μm)	328.6 (±99.4)	310.4 (±86.8)	354.2 (±110.9)	0.033*
Changes in CST(μm)	−93.4 (±160.3)	−131.3 (±141.6)	−39.9 (±171.5)	0.009*
Number of IVI	9.24 (±3.55)	9.51 (±3.68)	8.87 (±3.37)	0.421

BCVA: best corrected visual acuity, CST: central subfield thickness, HbA1C: glycosylated hemoglobin, LogMAR: logarithm of the minimum angle of resolution, IVI: intravitreal injection, PDR: Proliferative diabetic retinopathy, *P < 0.05.

### Factors significantly associated with the response to intravitreal ranibizumab

The results of logistic generalized estimating equations (GEE) model analyses of relevant factors associated with the response to ranibizumab showed that age (p = 0.003) and BCVA at baseline (p = 0.001) were significantly associated with the responders to intravitreal ranibizumab therapy. Patients with younger age had better response to the treatment. In addition, eyes with worse baseline BCVA had a wider range of improvement in BCVA after treatment. However, gender (p = 0.395), HbA1c (p = 0.805), baseline CST (p = 0.556) and PDR (p = 0.545) were not associated with the response to treatment (Table 2).

**Table 2:** Logistic GEE models for impact of clinical factors on the response to intravitreal ranibizumab therapy (responder/non-responder).

	Regression Coefficient (B)	Standard Error (S.E.)	P value
Age (years)	−0.103	0.034	0.003*
Gender	0.473	0.557	0.395
HbA1c (%)	−0.054	0.219	0.805
Baseline BCVA (LogMAR)	2.685	0.772	0.001*
Baseline CST (μm)	0.001	0.002	0.556
PDR	0.366	0.604	0.545

BCVA: best corrected visual acuity, CST: central subfield thickness, HbA1C: glycosylated hemoglobin, PDR: Proliferative diabetic retinopathy, *P < 0.05.

### Prognostic factors for visual outcome after intravitreal ranibizumab

In terms of the visual outcome, Table 3 and Table 4 illustrates the clinical factors affecting the changes in BCVA and final BCVA after treatment, respectively. In all subjects, GEE models revealed that age (p = 0.001 for both), baseline BCVA (p = 0.022, and p = 0.036, respectively) and number of intravitreal injections (p = 0.035 for both) were significantly associated with the changes in BCVA and final BCVA after treatment. Patients with younger age, better BCVA at baseline had better visual outcome. However, there was no association between HbA1c with the changes in BCVA or final BCVA (p = 0.086, and p = 0.085, respectively). In subgroup analyses, HbA1c (p = 0.013 for both) and baseline CST (p = 0.019 for both) were significantly associated with the changes in BCVA and the final BCVA after the treatment in the responder group. The patients with better glycemic control had better visual outcome after treatment. In addition, baseline BCVA was also associated with the changes in BCVA (p = 0.028), but not with the final BCVA (p = 0.254). Nonetheless, age (p = 0.553, and p = 0.555, respectively) and number of intravitreal injections (p = 0.053, and p = 0.055, respectively) were not associated with the changes in BCVA and the final BCVA after treatment. In the non-responder group, both the presence of PDR (p = 0.019 for both) and baseline CST (p = 0.002 for both) were significantly associated with the changes in BCVA and the final BCVA after treatment. Patients with PDR had worse visual outcome after treatment in the non-responder group. Furthermore, the final BCVA was also significantly influenced by the baseline BCVA (p = 0.001).

**Table 3:** GEE models for the impact of clinical factors on the changes in BCVA after treatment.

	All subjects		Responder group		Non-responder group	
	B (S.E.)	P value	B (S.E.)	P value	B (S.E.)	P value
Age (years)	−0.016 (0.005)	0.001*	−0.002 (0.004)	0.553	−0.008 (0.005)	0.070
HbA1c (%)	−0.056 (0.033)	0.086	−0.060 (0.024)	0.013*	0.019 (0.027)	0.478
Baseline BCVA (LogMAR)	0.520 (0.227)	0.022*	0.657 (0.300)	0.028*	0.250 (0.231)	0.279
Baseline CST (μm)	−0.002 (0.001)	0.063	−0.002 (0.001)	0.019*	−0.002 (0.001)	0.002*
PDR	0.005 (0.097)	0.959	0.083 (0.067)	0.212	−0.143 (0.061)	0.019*
No of IVI	−0.070 (0.033)	0.035*	−0.061 (0.032)	0.053	−0.017 (0.019)	0.389

B (S.E.): regression coefficient (standard error), BCVA: best corrected visual acuity, CST: central subfield thickness, HbA1C: glycosylated hemoglobin, IVI: intravitreal injection, PDR: Proliferative diabetic retinopathy, *P < 0.05.

**Table 4:** GEE models for the impact of clinical factors on the final BCVA after treatment.

	All subjects		Responder group		Non-responder group	
	B (S.E.)	P value	B (S.E.)	P value	B (S.E.)	P value
Age (years)	0.016 (0.005)	0.001*	0.002 (0.004)	0.555	0.008 (0.005)	0.070
HbA1c (%)	0.057 (0.033)	0.085	0.060 (0.024)	0.013*	−0.019 (0.027)	0.481
Baseline BCVA (LogMAR)	0.478 (0.228)	0.036*	0.343 (0.301)	0.254	0.745 (0.233)	0.001*
Baseline CST (μm)	0.002 (0.001)	0.062	0.002 (0.001)	0.019*	0.002 (0.001)	0.002*
PDR	−0.005 (0.098)	0.962	−0.083 (0.067)	0.217	0.144 (0.061)	0.019*
No of IVI	0.070 (0.033)	0.035*	0.061 (0.032)	0.055	0.016 (0.020)	0.401

B (S.E.): regression coefficient (standard error), BCVA: best corrected visual acuity, CST: central subfield thickness, HbA1C: glycosylated hemoglobin, IVI: intravitreal injection, PDR: Proliferative diabetic retinopathy, *P < 0.05.

### Prognostic factors for anatomic outcome after intravitreal ranibizumab

With regard to the anatomic outcome after treatment, baseline CST (p < 0.001, p < 0.001 and p = 0.005, respectively) were significantly associated with the changes in CST after treatment in all subjects, responder and non-responder groups (Table 5). Nevertheless, the changes in CST was significantly influenced by age (p = 0.003) only in the responder group. Patients with younger age showed greater reduction in CST after treatment. The presence of PDR (p = 0.041) was found to be associated with less reduction in CST after treatment in non-responder group. Furthermore, there was no significant difference in the distribution of OCT types at baseline between the responder and non-responder groups (P = 0.896) (Table 6).

**Table 5:** GEE models for the impact of clinical factors on the anatomical outcome (the changes in CST) after treatment.

	All subjects		Responder group		Non-responder group	
	B (S.E.)	P value	B (S.E.)	P value	B (S.E.)	P value
Age (years)	−0.287 (0.945)	0.771	−2.411 (0.809)	0.003*	2.563 (2.175)	0.239
HbA1c (%)	2.394 (8.918)	0.788	6.312 (6.420)	0.326	−19.757 (17.542)	0.260
Baseline BCVA (LogMAR)	−31.220 (64.816)	0.630	−100.204 (85.381)	0.241	290.325 (208.219)	0.163
Baseline CST (μm)	−1.290 (0.217)	<0.001*	−1.370 (0.240)	<0.001*	−1.428 (0.507)	0.005*
PDR	5.480 (18.931)	0.772	−25.001 (18.770)	0.183	87.705 (42.859)	0.041*
No of IVI	−5.807 (10.834)	0.592	−24.841 (14.515)	0.087	15.098 (10.313)	0.143

B (S.E.): regression coefficient (standard error), BCVA: best corrected visual acuity, CST: central subfield thickness, HbA1C: glycosylated hemoglobin, IVI: intravitreal injection, PDR: Proliferative diabetic retinopathy, *P < 0.05.

**Table 6:** Optical coherence tomography types in diabetic macular edema at baseline between responders and non-responders.

	OCT types in diabetic macular edema			
	Sponge-like retinal swelling	Cystoid macular edema	Serous retinal detachment	Mixed type
Total number (94 eyes)	36 (38%)	33 (35%)	13 (14%)	12 (13%)
Responders (55 eyes)	20 (36%)	21 (38%)	7 (13%)	7 (13%)
Non-responders (39 eyes)	16 (41%)	12 (31%)	6 (15%)	5 (13%)

OCT: Optical coherence tomography. Chi-square test, P = 0.896

## Discussion

Several clinical trials have recently demonstrated that intravitreal anti-VEGF therapy for DME leads to improvement in both visual and anatomic outcomes at 1 to 3 years’ follow-up^10,20–26^. This study also affirms intravitreal ranibizumab therapy as a promising treatment for DME. However, not all patients with DME respond well to intravitreal ranibizumab treatment. In this study, we sought to investigate the factors influencing the visual outcome in patients with DME treated with ranibizumab. Patients with younger age showed better response to the treatment. In subgroups analyses, there were some differences between the responder and non-responder groups not only in the baseline characteristics but also in the prognostic factors. In the responder groups, patients were younger and had worse BCVA and thicker CST at baseline. With regard to prognostic factors, baseline CST was associated with the changes in BCVA, final BCVA and the changes in CST in both groups. Nonetheless, better glycemic control was associated with better visual outcome only in responder cases. In the non-responder group, the presence of PDR was associated with worse visual and anatomic outcomes.

Although the promising outcomes of anti-VEGF treatment for DME, there were still considerable variations in the clinical outcomes among patients. Several prognostic factors have been studied in several large randomized clinical trials (RCTs) by post hoc retrospective analysis, but there were no consensus results from these studies^15,16,19,27^. In general, better baseline BCVA was associated with better visual outcome^16,19,27^. Furthermore, poor baseline BCVA and young age appeared to have more visual gains after treatment^15,19^. Male patients tended to have better visual outcome^19^. In this study, we found the prognostic factors, including age, BCVA at baseline and number of intravitreal injections, showed a significant influence on the visual outcome for DME after intravitreal ranibizumab treatment in the data analysis from all subjects. Younger age, better BCVA at baseline and more intravitreal injection were associated with better final visual outcome. Nonetheless, no gender difference in visual outcome was noted. Furthermore, we separated the patients into the responder and non-responder groups according to the visual outcome. In the logistic GEE analysis, age and baseline BCVA were the predictors for the responder after the treatment. Younger age and worse baseline BCVA were associated with better visual gains (responder group). These results were in line with the findings from previous studies.

Previous studies have defined the treatment response according to anatomical^15,28–30^ or visual outcomes^29,30^. The definition of visual response was similar across studies (BCVA gain more than one line or LogMAR change <0). However, the definition of anatomical response was inconsistent among studies. For example, Bresseler *et al*.^15^ divided all subjects into 4 categories: “early and consistent”, “early but inconsistent”, “slow and variable”, and the “non-responder” groups according to 20% reduction in OCT changes during the first treatment year. Koyanagi *et al*.^28^ defined the “responders” as those with more than 25% decrease in CST at 3 months after treatment. In addition, several studies revealed that some patients had delayed anatomic response after continuing anti-VEGF treatment for DME^12,30–32^, and early anatomic response might not be fully consistent with final anatomic outcomes. It also found that a wide range of visual acuities may exist for any degree of macular thickness, suggesting some discordance between visual and anatomic outcomes after anti-VEGF treatment for DME^12,13,31^ Moreover, BCVA gain is important for patient’s quality of life. Therefore, we define the treatment response according visual outcomes similar to some previous reports^29,30^.

A further subgroup analysis of the prognostic factors, there were significant differences between the responder and non-responder groups. In the responder group, HbA1c and baseline CST were significant predictors to the changes in BCVA and final BCVA. Baseline BCVA was associated with only the changes in BCVA, but not the final VA. This probably can be explained by “the ceiling effect”, i.e. patients had return to normal vision despite the differences in baseline BCVA. In the non-responder group, thicker baseline CST and the presence of PDR were likely to have poor visual outcome. Baseline BCVA was significantly related to final BCVA, but not the changes in BCVA. Nevertheless, there was no significant association between age and the visual outcome in the both groups. For the anatomic outcome, thicker baseline CST tended to have greater reduction in CST in all subjects and subgroups analysis. This finding was in agreement with the results in the Diabetic Retinopathy Clinical Research Network study that baseline CST is the strongest predictor of anatomic outcome^15^. Furthermore, younger age showed significant greater reduction in CST only in the responder group. The presence of PDR had significantly less changes in CST after treatment in non-responder groups. This study revealed not only different baseline clinical characteristics, but also the different prognostic factors for visual and anatomic outcomes between the responder and non-responder groups. Dabir *et al*.^33^ have also reported differential systemic gene expression profiles between the responder and non-responder to bevacizumab treatment for DME. They found the expression of IL8 is much higher in non-responder cases and suggest that inflammation may have a major role in distinguishing the responder and non-responder groups. Thus, the differences in clinical characteristics between two groups might be related to different proportions of contribution of the underlying pathological mechanisms for DME, such as angiogenesis and inflammation, between them.

As for the administration protocol, patients were treated with three monthly loading injections, and then followed approximately every 4–6 weeks by further injections as required, which was similar to the treatment protocol in the RESTORE study^11^. Although more intravitreal injections seemed to be associated with better final visual outcomes in the analysis with all subjects, no significant influence of injection number on the final visual outcomes was found in the subgroup analysis of responders and non-responders. Moreover, less number of intravitreal injections in the 12 months was noted in the non-responders (8.87) compared to responders (9.51), whereas there was no statistical difference between them. The plausible explanation could be the poorer compliance of some non-responder patients. Due to the retrospective nature of this study, some non-responder patients with limited visual response after 3 monthly injections might postpone the visit or the treatment time, which resulted in slight less number of injections in the first year.

We found patient age was related with the visual outcome of DME after intravitreal ranibizumab treatment, and younger patients appeared to respond more favorably to the treatment. This is consistent with the results in previous studies^15,19^. Bressler *et al*.^15^ found that younger age was associated with higher VA gains after intravitreal ranibizumab treatment for DME. Similar results had been revealed in treating patients with neovascular age-related macular degeneration by intravitreal ranibizumab^34,35^. Sophie *et al*.^19^ suggested that the macula in young patients might be more tolerable to fluid accumulation without incurring loss of visual potential. In this study, age was revealed to be a strong predictor for responder to the treatment. In addition, age was no longer associated with visual outcome in the subgroups analysis, suggesting that the influence of age was related to the response to intravitreal ranibizumab therapy.

Glycosylated hemoglobin (HbA1c) is a biomarker for monitoring the levels of blood glucose among diabetic patients and indicates the average blood glucose levels over the previous three months^36^. Multiple large epidemiologic studies have shown that elevated HbA1c levels confers an increased risk of developing DME^2,5,37,38^. The influence of HbA1c on the visual outcome of anti-VEGF treatment for DME was controversial in previous studies^17,18,39–42^. Matsuda *et al*.^17^ found that a significant improvement in BCVA in patients with HbA1c ≤ 7.0% after anti-VEGF therapy, whereas a less robust improvement in BCVA was observed in patients with HBA1c > 7.0%. In addition, patients with high HbA1c level were less likely to have VA improvement after bevacizumab treatment^41^. Nevertheless, Singh *et al*.^39^ revealed vision improvement with ranibizumab was not affected by systemic factors, for example, HbA1c, renal function and blood pressure. A post hoc analysis of the RIDE/RISE Trials showed that the VA improvement, remission of macular edema, and improvement in DR severity score with ranibizumab treatment appeared to be independent of HbA1c at baseline^18^. Interestingly, HbA1c showed significant association with final visual outcome and the changes in BCVA only in the responder group, but not in the non-responder group in our study. Patients with lower HbA1c level tended to have better visual outcome after the treatment, suggesting that blood sugar control still plays a role in visual outcome of anti-VEGF treatment for DME.

In this study, the presence of PDR had a negative influence on both the visual and anatomic outcomes in the non-responder cases. Previous studies have also shown that eyes with PDR or prior PRP had less visual acuity benefits after anti-VEGF treatment^15,19^. These findings suggested that eyes with more severe DR may be more likely to have ischemic damage to retina, especially the macula, which precluded greater improvement in the visual outcome. Although there was no difference in the number of eyes with PDR between two groups, the impact of PDR on the treatment outcomes was not observed in the responder cases. The reason for this may be related to the extent of retinal ischemic damage. The responder case may have less extent of retinal ischemic damage than the non-responder case, and therefore result in a greater potential for visual improvement. Moverover, these results could in part be explained by the the VEGF-positive feedback loop which was proposed by Campochiaro *et al*.^43^. Although Ranibizumab neutralizes VEGF and interrupts this feedback loop to reduce DME, hyperglycemia-induced damage to retinal vessels could be continued progression in patient with poorer blood sugar control and lead to further retinal vessels occlusion and poorer visual outcomes. Hence, a significant effect of HbA1c on visual outcomes after treatment was noted in the responders. However, when the progression of retinal vessels occlusion achieved to certain threshold levels or more extensive areas, ranibizumab could no longer interrupt this feedback loop and resulted in poorer visual outcomes even after multiple treatment. Therefore, eye with the presence of PDR in the non-responders tended to have poorer visual outcomes in our results.

In the past reports^15–19^, they performed the detailed analysis using all the subjects together to find the prognostic factors for DME treated with ranibizumab. Channa *et al*.^16^ compared the clinical characteristics between poor and better visual outcome groups and further analyzed the factors only in the poor visual outcome groups. In this study, we also found the different clinical characteristics between responder and non-responders, such as age, baseline BCVA and CST. Hence, we performed the subgroup analyses to investigate whether the prognostic factors were different between these two groups. Our results showed a borderline significant trend that the Influence of HbA1c on the visual outcomes of ranibizumab for DME in the analysis of all subjects. In the subgroup analysis, HbA1c showed a significant effect on visual outcomes in the responders, and the presence of PDR was likely to have poor visual outcome in the non-responder. Due to the heterogeneity in the response to ranibizumab for DME, subgroup analysis may help to disentangle some inconclusive results from the analysis of all subjects and provide further information regarding those patients who benefit the most after treatment in each group. In this study, we analyzed the prognostic factors not only for non-responders, but also for responders, it would be important in the clinic practice to know what factors can facilitate the better visual outcomes for either group.

Limitations of the current study include its retrospective nature, potential selection bias (HbA1c < 10% and baseline BCVA), the relatively short follow-up time, limited information on duration of DME and the possible fluctuation of HbA1C level during the treatment. Furthermore, small sample size may have attenuated the statistical power for detecting differences between the groups. To better predict the treatment outcomes of intravitreal anti-VEGF for DME, further prospectively designed studies with larger sample size are needed.

In conclusion, this study showed a significant benefit in both anatomic and functional outcomes with intravitreal ranibizumab therapy for DME. Younger age, better BCVA at baseline were associated with better final visual outcome. The anatomic outcome was significantly influenced by the baseline CST. In addition, the presence of PDR had worse anatomic and visual outcomes in non-responder groups. Patients with lower HbA1c level tended to have better visual outcome after the treatment in the responder group. This study revealed the importance of the blood sugar control for the patients with DME receiving intravitreal anti-VEGF therapy.

## Methods

A retrospective consecutive case review was performed for patients with DME who received intravitreal ranibizumab treatment from January 2014 to December 2015 at Changhua Christian Hospital. The Institutional Review Board of Changhua Christian Hospital in Changhua, Taiwan, approved the study protocol and waived the requirement of written informed consent. All methods were performed in accordance with the relevant guidelines and regulations.

### Patients

All Patients with DME underwent complete ophthalmologic examination, including BCVA, intraocular pressure, fundus color photography, fundus fluorescein angiography, and spectral-domain optical coherence tomography (SD-OCT). The inclusion criteria included (1) age 20 years or older, (2) center-involving DME, (3) BCVA in the study eye between 0.05 to 0.5 measured by Snellen chart, (4) DME with CST of 300 μm or more measured by SD-OCT, and (5) serum HbA1c less than 10%. The exclusion criteria were (1) prior vitreoretinal surgery, (2) intraocular corticosteroids, anti-VEGF or macular grid or micropulse laser treatment before initiation of anti-VEGF therapy, (3) presence of significant media opacity that would limit vision recovery (e.g., significant cataract, vitreous hemorrhage), (4) presence of any retinal disease other than DR (e.g., macular degeneration, retinal vascular occlusions), (5) co-existing vitreomacular traction or epiretinal membrane as determined by SD-OCT, and (6) less than 1-year follow-up from initial injection.

### Intravitreal Anti-VEGF Injection

All patients underwent intravitreal ranibizumab (0.5 mg/0.05 mL) injection for DME under aseptic conditions. Given the retrospective nature of this study, the treatment regimen was at the treating physicians’ discretion. Typically, patients were treated using an initial series of three monthly loading injections, and then followed approximately every 4–6 weeks by further injections as required. Indications for retreatment included center-involving DME with CST more than 300 μm or no further improvement in BCVA as compared with 2 preceding visits. Micropulse laser therapy was performed as rescue therapy during the treatment period at the treating physician’s discretion.

The data collected included the patient’s age, gender, BCVA and CST at baseline, 3,6,12 months and at final visit after intravitreal ranibizumab treatment, and serum HbA1c at baseline. Furthermore, all eyes were classified into two groups (responder, non-responder) according to the final visual outcome after intravitreal ranibizumab treatment for DME. The responder group was defined as the patients who had a final BCVA improvement of one or more lines compared with baseline BCVA, and the non-responder group was defined as patients having no changes or a reduction in final BCVA^44^. The clinical factors, including age, gender, baseline BCVA, baseline CST by SD-OCT, HbA1c, and number of injections, were investigated for their effect on the clinical outcomes after intravitreal ranibizumab treatment for DME in responder and non-responder groups, respectively. Moreover, we categorized all OCT images at baseline into 4 types: sponge-like retinal swelling, cystoid macular edema, serous retinal detachment and mixed types (combined either two types above) according to the classification by Otani *et al*.^45^, the relationship between the OCT morphology in DME and the response for intravitreal ranibizumab was also investigated.

### Statistical analysis

All statistical analyses were performed using SPSS for Windows, version 13.0 software (SPSS Science, Chicago, IL, USA). Snellen BCVA was converted to LogMAR notation for analysis purposes. To take into account the correlations between two eyes of the same patient, GEE models were fitted to the data^46^. For comparing the difference in BCVA and CST between baseline and after treatment, GEE models were used with the laterality of eye and repeated measures as within-subject variables. The differences in age, gender, baseline and final BCVA, baseline and final CST by SD-OCT, HbA1c, and number of injections between anti-VEGF responder and non-responder groups were also evaluated using GEE models with the laterality of eye as within-subject variable and the response to ranibizumab as predictive factor. Furthermore, a logistic GEE model was applied to investigate the factors associated with the response to ranibizumab with the responder/non-responder as dependent variable. GEE models were used to determine whether changes in BCVA and CST after treatment varied according to the clinical characteristics in all subjects, responder and non-responder groups, respectively. In addition, Pearson chi-square tests were used to examine if there is any difference in OCT types between responders and non-responders. A P-value < 0.05 was considered as statistically significant.

## Data Availability

The datasets generated during and/or analysed during the current study are not publicly available due to the risk of violating patient privacy but are available from the corresponding author on reasonable request.
